# Effect of Phase Transformation on Stress Corrosion Behavior of Additively Manufactured Austenitic Stainless Steel Produced by Directed Energy Deposition

**DOI:** 10.3390/ma14010055

**Published:** 2020-12-24

**Authors:** Tomer Ron, Ohad Dolev, Avi Leon, Amnon Shirizly, Eli Aghion

**Affiliations:** Department of Materials Engineering, Ben-Gurion University of the Negev, Beer-Sheva 8410501, Israel; dolev.ohad@gmail.com (O.D.); avileon12@gmail.com (A.L.); a.shirizly@gmail.com (A.S.); egyon@bgu.ac.il (E.A.)

**Keywords:** additive manufacturing, direct energy deposition, wire arc additive manufacturing, 316L stainless steel, stress corrosion

## Abstract

The present study aims to evaluate the stress corrosion behavior of additively manufactured austenitic stainless steel produced by the wire arc additive manufacturing (WAAM) process. This was examined in comparison with its counterpart, wrought alloy, by electrochemical analysis in terms of potentiodynamic polarization and impedance spectroscopy and by slow strain rate testing (SSRT) in a corrosive environment. The microstructure assessment was performed using optical and scanning electron microscopy along with X-ray diffraction analysis. The obtained results indicated that in spite of the inherent differences in microstructure and mechanical properties between the additively manufactured austenitic stainless steel and its counterpart wrought alloy, their electrochemical performance and stress corrosion susceptibility were similar. The corrosion attack in the additively manufactured alloy was mainly concentrated at the interface between the austenitic matrix and the secondary ferritic phase. In the case of the counterpart wrought alloy with a single austenitic phase, the corrosion attack was manifested by uniform pitting evenly scattered at the external surface. Both alloys showed ductile failure in the form of “cap and cone” fractures in post-SSRT experiments in corrosive environment.

## 1. Introduction

Additive manufacturing (AM) has been considered to be a promising technology for producing a variety of complex components in a relatively short time [1,2,3,4,5,6]. Traditional AM processing of metals mainly focuses on powder bed fusion (PBF) methods such as selective laser melting (SLM) and electron beam melting (EBM) [7,8,9,10]. However, PBF technologies are relatively expensive due to the high cost of raw material, high energy consumption and relatively low deposition rate. In addition, the size of the printed component is limited and depends on the printing cell dimension. The inherent disadvantages of PBF technologies highlight the need to use more affordable AM methods such as the wire arc additive manufacturing (WAAM) process. Comparatively, proven PBF technologies can produce a deposition rate of 0.1 kg/h, while the deposition rate of WAAM is about 10 kg/h [11,12,13]. In addition, the use of relatively inexpensive wires as raw materials and an electric arc as the energy source can reduce the cost of the printing process by 80% compared to PBF [14,15]. Furthermore, the dimensions of components produced by WAAM are almost unlimited [16]. It should be pointed out that WAAM process can be also implemented using computer numerical control (CNC) systems [17]. The almost unlimited dimensions of WAAM is due to the fact that the printing process can be performed by an external robot that is free to move in all directions [18,19,20]. However, there are some limitations related to the WAAM process compared to PBF technology. This includes relatively increased surface roughness, and limited capabilities to produce complex structures. Currently, most of the research activities related to the WAAM process have focused on optimizing the printing parameters and residual stresses status [21,22,23,24,25], with very limited attention paid to the corrosion performance of the obtained components. This study mainly aims at evaluating the effect of phase transformation on stress corrosion behavior of additively manufactured austenitic stainless steel in the form of 316L alloy produced by the WAAM process. For reference consideration, the obtained stress corrosion behavior was compared to its counterpart wrought alloy AISI 316L. The general corrosion performance was evaluated in terms of potentiodynamic polarization and electrochemical impedance spectroscopy (EIS) analyses all in 3.5% NaCl solution.

## 2. Materials and Methods

**Figure 1:**
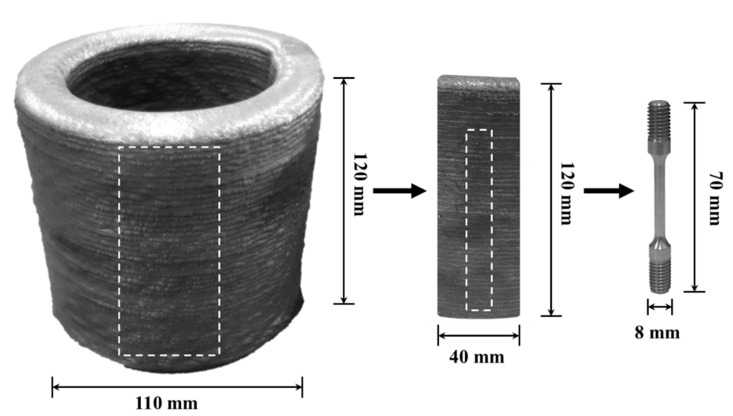
General appearance of the cylindrical component along with a tension specimen obtained by the WAAM process.

The tested specimens were machined from a hollow cylindrical component produced by the WAAM process with a conventional metal inert gas (MIG) welding system, using austenitic 316L stainless steel welding wire, as shown in Figure 1 [20] along with a tension specimen. No additional heat treatment was applied and all tested were carried out in as-build conditions. The welding wire diameter was 1.14 mm and the dimensions of the cylindrical component were: 120 mm height, 55 mm mid-wall radius and 15 mm wall thickness. The wire deposition was implemented using a Cloos Rotrol V7.13 robot (CLOOS, Haiger, Germany) that was integrated with a welding head system. The welding pathway was controlled by a computer-aided design (CAD) model. The welding parameters in terms of the deposition process included: wire feed rate of 6.1 m/min, electrical current of 210 A, voltage of 23.9–24.1 V, pulse frequency of 120 Hz, and robot deposition speed of 14 cm/min. The deposition process was carried out under a protective gas atmosphere composed of 98% Argon and 2% Oxygen. The printing parameters were selected based on regular welding conditions used by MIG-welding of steels.

Microstructure analysis was carried out by scanning electron microscopy (SEM-JEOL 5600, JEOL Ltd., Tokyo, Japan) [26] equipped with an EDS sensor (Thermo Fisher Scientific, Waltham, MA, USA) for phase chemical composition detection. The metallographic preparation included polishing up to 0.04 µm and subsequent etching with HNO_3_ (70%) 20 mL, HCl (32%) 45 mL and ethanol 25 mL for 1.5 min. The presence of a secondary phase in the form of ferrite (BCC) was evaluated by X-ray diffraction analysis using a RIGAKU-2100H X-ray diffractometer (Rigaku Corporation, Tokyo, Japan) with CuKα [27]. The diffraction parameters were 40 KV/30 mA and a scanning rate of 2°/min.

The corrosion performance was examined in terms of electrochemical analysis by potentiodynamic polarization and EIS using a Bio-Logic SP-200 potentiostat (BioLogic Science Instruments, Seyssinet-Pariset, France) equipped with EC-Lab software v11.18. Both analyses were implemented using a standard three-electrode cell with saturated calomel (SCE) as a reference electrode and Platinum as an auxiliary electrode. The potentiodynamic polarization scanning rate was 0.5 mV/s, and the EIS examination was performed between 10 kHz and 0.015 Hz at a 10 mV amplitude signal. The preparation procedure of the samples for the electrochemical testing included cleaning in an ultrasonic bath for 5 min, washing with alcohol and drying in air. The stress corrosion behavior in terms of SSRT was examined according to ASTM G129-00 standard [28], using Cormet C-76 apparatus (Cormet Testing Systems, Vantaa, Finland) [29,30]. The dimensions of the SSRT test samples were: gauge length 25.4 mm and cylindrical cross section 11.4 mm^2^. The SSRT strain rates were: 2.5 × 10^−7^, 2.5 × 10^−6^ and 2.5 × 10^−5^ s^−1^. All the corrosion tests were carried out in 3.5% NaCl solution at ambient temperature (25 °C).

## 3. Results

The chemical composition of the welding wire, printed alloy and counterpart AISI 316L stainless steel alloy are shown in Table 1. This reveals that the composition of the printed alloy was in line with the composition of the welding wire and quite similar to that of the counterpart AISI alloy in terms of main alloying elements and carbon content.

**Figure 2:**
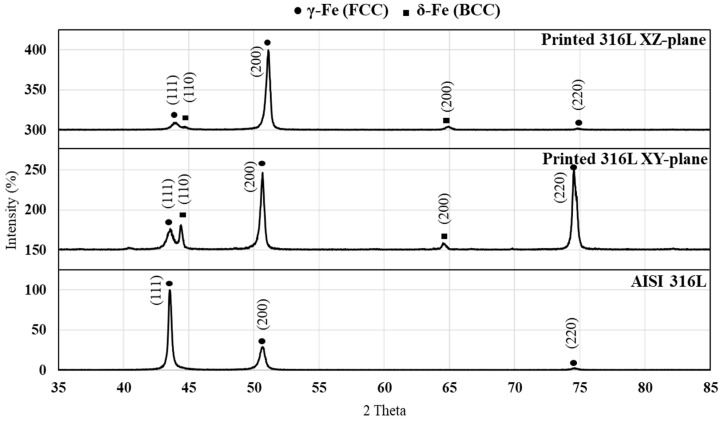
X-ray diffraction analysis of printed alloy and AISI 316L alloy.

X-ray diffraction analysis of the printed alloy and its counterpart AISI 316L are shown in Figure 2. This reveals that the printed alloy was composed from an austenitic matrix (γ-Fe) and a secondary ferritic phase (δ-Fe), as expected from a regular 316L printed alloy [31] while the stock material (wire) has one austenitic phase. In parallel, the counterpart AISI 316L was composed from only an austenitic phase. In addition, significant differences were obtained between the XY-plane (building direction 0°) and XZ-plane (building direction 90°) of the printed alloy in terms of peak intensity. This can be attributed to the epitaxial characteristics of the AM process that display a preferred orientation of the solidification process. The calculated lattice parameter of the γ-Fe and δ-Fe related to the printed alloy were 2.88 and 3.59 Å, respectively, which basically comes in line with the parameters found in the literature: 2.86 Å (PDF 006–096) and 3.59 Å (PDF 33–0397). In the case of the counterpart AISI 316L, the calculated lattice parameter was 3.59 Å, as expected. Furthermore, it should be pointed out that, in contrast to the microstructure of 316L obtained by the WAAM process as presented by X. Chen et al. [25,32], no σ-phase was observed in this study.

**Figure 3:**
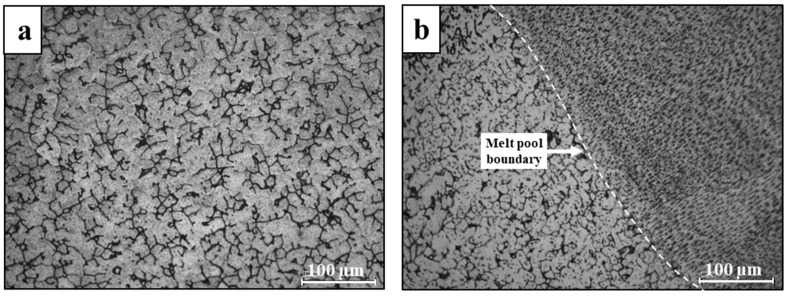
Typical microstructures of printed 316L stainless steel obtained by optical microscopy (**a**) XY-plane, (**b**) XZ-plane.

**Figure 4:**
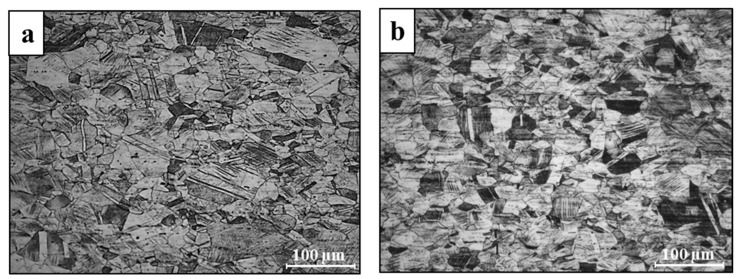
Typical microstructures of AISI 316L stainless steel (**a**) longitudinal cross section, (**b**) transvers cross section.

Typical microstructure of printed 316L stainless steel in the XY- and XZ-planes is shown in Figure 3 while the microstructure of its counterpart AISI 316L alloy is introduced in Figure 4. The microstructure of the printed alloy was composed from an austenitic matrix and a secondary ferritic phase at the grain boundaries. The ferritic dendrites in the XY-plane present an anisotropic morphology, while the XZ-plane introduces an epitaxial solidification characteristic. In parallel, the counterpart AISI 316L was composed of a single austenitic phase, as expected from conventional 316L stainless steel [33]. The two-phase microstructure of the printed alloy was formed due to the high solidification rate of the AM process. This can also be explained in terms of the Fe-Cr-Ni phase diagram [34] operating under high cooling rate conditions. In addition, the inherent re-heating effect of the AM process produces a non-equilibrium microstructure that displays the presence of a primary δ ferrite phase [25].

**Figure 5:**
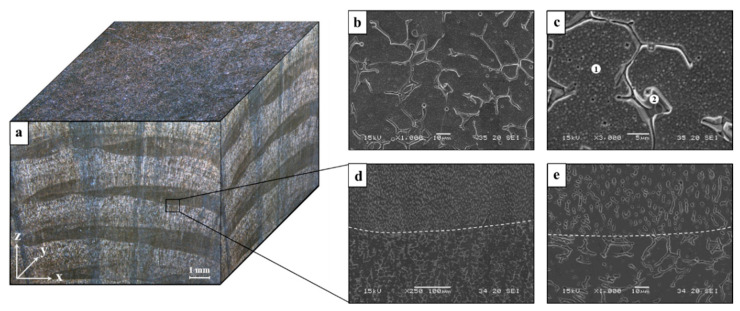
Macrostructure and corresponding microstructures of printed 316L alloy obtained by stereo microscope and SEM (**a**) general macrostructure, (**b**,**c**) microstructure in the XY-plane, (**d**,**e**) microstructure in the XZ-plane.

The general macrostructure of the printed alloy in 3D is shown in Figure 5, along with the corresponding close-up microstructures and spot chemical analysis. This clearly reveals that the microstructure in the XY-plane was quite uniform compared to the non-uniform structure in the XZ-plane that relates to the preferred orientation of the solidification course. In addition, the melt pool boundaries shown in Figure 5d,e clearly illustrate the epitaxial nature of solidification in the XZ-plane. The spot chemical analyses at points 1 and 2 (Table 2) disclose the typical compositions of austenite and ferrite phases, respectively. These compositions reflect the relatively increased amount of Ni and reduced content of Cr in the austenitic phase and vice versa in the ferritic phase.

**Figure 6:**
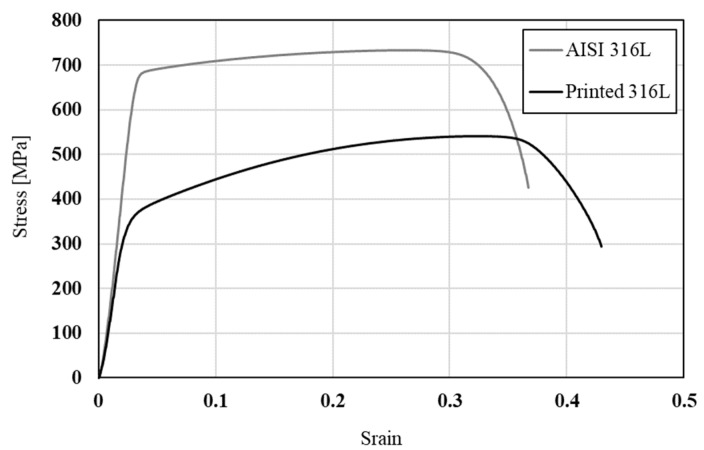
Typical stress-strain curves of printed 316L alloy and counterpart AISI 316L.

The mechanical properties of printed 316L and its counterpart AISI 316L in terms of tensile strength, yield strength, elongation and hardness are shown in Table 3 along with their typical stress-strain curves shown in Figure 6. This reveals that the strength of the printed alloy was relatively reduced, while its ductility was increased compared to the counterpart AISI 316L.

**Figure 7:**
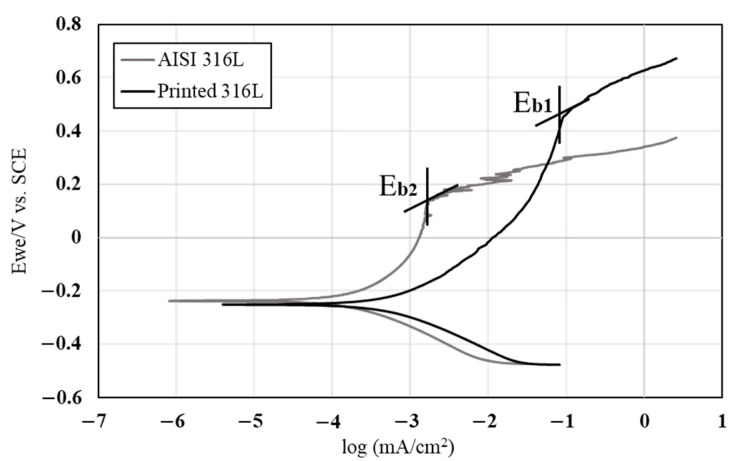
Potentiodynamic polarization analysis of printed 316L alloy and counterpart AISI 316L in 3.5% NaCl solution.

**Figure 8:**
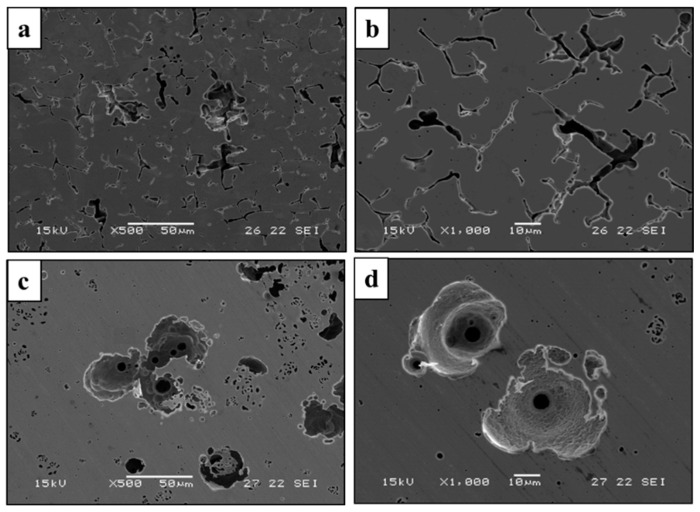
(**a**,**b**) Close-up views of the corrosion attack at surface of printed alloy, (**c**,**d**) close-up views of the corrosion attack at surface of counterpart AISI alloy.

The corrosion resistance of the printed and counterpart AISI 316L in terms of potentiodynamic polarization analysis is shown in Figure 7. Although the polarization curve of the printed alloy was relatively shifted to higher corrosion currents, which reflects reduced corrosion resistance [35,36], its break potential (*E*_b1_) was relatively higher compared to the AISI 316L (*E*_b2_), which reflects an improved passivation process. Altogether, the corrosion rate of the printed alloy in terms of Tafel extrapolation was excellent, and quite similar to the counterpart alloy as shown in Table 4 (0.005 vs. 0.001 mmpy, respectively). In addition, as expected from metals having an active-passive transition, both alloys showed localized corrosion attack. In the case of the printed alloy the localized corrosion attack was concentrated at the boundaries between the austenite and ferrite phases (Figure 8a,b) [37]. The corrosion attack in the counterpart AISI 316L was in the form of pitting corrosion (Figure 8c,d) that was evenly scattered on the external surface. The counterpart alloy presented typical pitting morphology for AISI 316L, as well as typical corrosion potential, breakdown potential and corrosion current [38].

**Figure 9:**
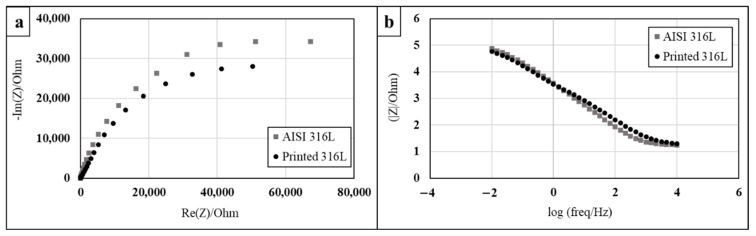
Electrochemical impedance spectroscopy analysis of printed and AISI 316L in 3.5% NaCl solution: (**a**) Nyquist diagram, (**b**) Bode diagram.

**Figure 10:**
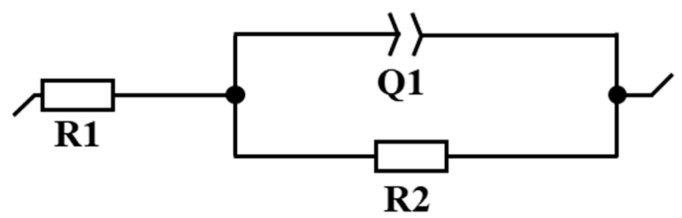
Electrical equivalent circuits for the EIS analysis shown in Figure 9.

The corrosion performance of printed and AISI 316L obtained by EIS analysis are shown in Figure 9. The Nyquist diagrams of both alloys (Figure 9a) in terms of curve radius, which represents the surface corrosion resistance, were quite similar. This similarity was also maintained by the Bode magnitude diagram (Figure 9b) that introduces the solution resistance. The related electrical equivalent circuit and corresponding fitting parameters (R1-solution resistance, R2 and Q1-capacitor) [39,40] are introduced in Figure 10 and Table 5, respectively. Altogether, the EIS analysis clearly indicates that the corrosion resistance of printed and counterpart alloys was quite similar.

**Figure 11:**
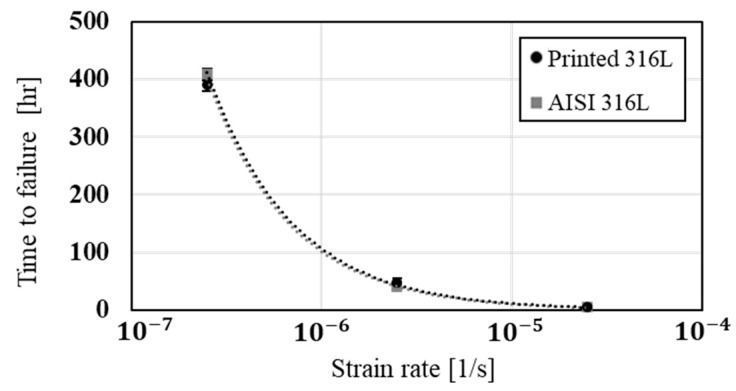
The effect of strain rate on time to failure of 316L produced by WAAM process in comparison with conventional wrought alloy AISI 316L.

**Figure 12:**
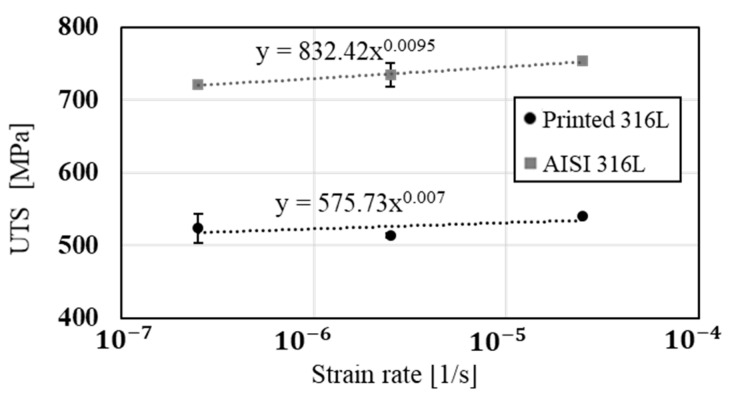
Ultimate tensile strength (UTS) versus strain rate of 316L produced by WAAM process compared to its counterpart wrought alloy AISI 316L.

**Figure 13:**
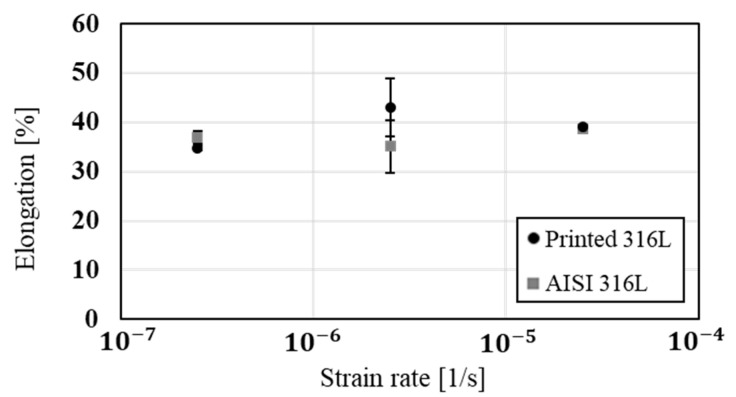
Ductility in terms of elongation versus strain rate of 316L produced by WAAM process compared to wrought alloy AISI 316L.

**Figure 14:**
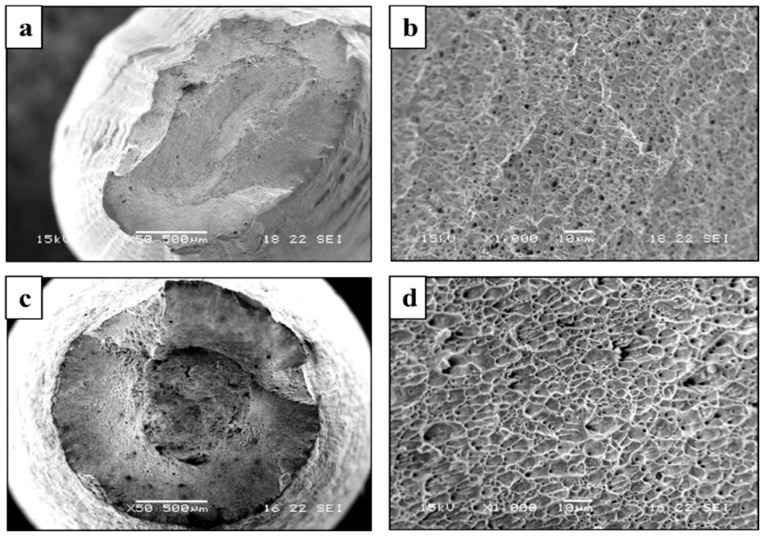
Fracture surface after SSRT at a strain rate of 2.5 × 10^−7^ (1/S) in 3.5% NaCl solution. (**a**,**b**) printed alloy, (**c**,**d**) counterpart AISI 316L.

The stress corrosion behavior of the printed and counterpart AISI 316L in terms of SSRT in 3.5% NaCl solution, are shown in Figure 8, Figure 9 and Figure 10. Although the stress corrosion performance of the two alloys was similar, as reflected by nearly equal time to failure vs. strain rate (Figure 11), the two alloys maintain their inherent UTS and elongation properties (Figure 12 and Figure 13, respectively). This similarity could also be seen by the fitting equations $\;(\sigma_{\mathrm{UTS}}=C\times {\dot{\varepsilon }}^{m})$ of UTS vs. strain rate According to these fittings, the stain rate sensitively factors (*m*) of the two alloys were very close: 0.007 and 0.009 for the printed and AISI 316L, respectively. Fractography analysis of the two alloys (Figure 14a–d) clearly demonstrates that both alloys showed ductile failure behavior in the form of “cap and cone” fractures, as expected from 316L stainless steel alloy.

## 4. Discussion

In spite of the differences between the microstructure and mechanical properties of WAAM 316L alloy and its counterpart AISI 316L, their corrosion performance in 3.5% NaCl solution was quite similar. This was strongly supported by the results of potentiodynamic polarization, EIS and stress corrosion analysis in terms of SSRT examination. Nevertheless, the corrosion mechanism of the two alloys was slightly different. This was clearly demonstrated by the surface corrosion attack shown in Figure 8. According to this figure, the localized corrosion attack in the printed alloy was mainly located at the interface between the austenitic matrix and the secondary ferritic phase (Figure 8a,b). This was mainly attributed to the relatively reduced corrosion resistance of the ferritic phase compared to the austenitic phase, which can induce micro-galvanic corrosion. In the case of the counterpart AISI 316L, the localized corrosion attack was in the form of pitting corrosion that was uniformly scattered on the surface (Figure 8c,d).

Regarding the effect of strain rate on UTS and elongation under a corrosive environment, both the printed and the counterpart AISI 316L displayed a similar response, according to their inherent mechanical properties. The similarity in their stress corrosion resistance was demonstrated by nearly equal time to failure at a slow strain rate of 2.5 × 10^−7^ s^−1^ (Figure 11), where the environmental effect was most dominant. This similarity was also manifested by the fractography analysis of the two alloys (Figure 14a–d) that clearly showed ductile failure characteristics in the form of “cap and cone” fractures, as can be expected from 316L stainless steel alloy [41].

As a final remark it should be pointed out that the similar corrosion performance of printed WAAM 316L alloy and its counterpart AISI 316L in 3.5% NaCl solution cannot be simply extrapolated to any different environment. This is mainly due to the inherent differences between the microstructure of the two alloys that can affect their corrosion behavior primarily in a more aggressive environment.

## 5. Conclusions

The stress corrosion behavior of additively manufactured austenitic stainless steel (316L alloy) produced by WAAM process in terms of stress corrosion susceptibility and electrochemical performance was similar to that of its counterpart wrought alloy. This similarity was obtained in spite of the inherent differences in microstructure and mechanical properties of the two alloys. The corrosion attack in the printed alloy was mainly located at the interface between the austenitic matrix and the ferritic phase, while that of the counterpart alloy composed of a single austenite phase was in the form of pitting corrosion uniformly scattered on the surface. The fractography analysis of the two alloys in post-SSRT experiments revealed that both alloys showed ductile failure in the form of “cap and cone” fractures, as expected from austenitic stainless steel.

## Figures and Tables

**Table 1:** Chemical composition (wt.%) of welding wire, printed stainless steel part and counterpart AISI 316L.

Material	C	Mn	Si	S	P	Cr	Ni	Cu	Mo	Co	N	Fe
Welding Wire	0.013	1.97	0.51	0.0010	0.018	19.22	11.50	0.15	2.42	0.07	0.092	Bal.
Printed 316L	0.024	1.85	0.44	0.0007	0.020	19.21	11.62	0.09	2.48	0.26	0.052	Bal.
AISI 316L	0.021	1.59	0.40	0.02	0.037	16.58	10.07	0.44	2.04	0.19	0.078	Bal.

**Table 2:** Spot chemical analysis of printed 316L at points 1 and 2 (Figure 5c) by EDS in wt.%.

Test Point	C	Mn	Si	Cr	Ni	Mo	Fe
Point 1	0.39	2.3	0.41	17.34	12.08	2.02	Bal.
Point 2	0.25	2.12	0.39	24.86	5.43	4.17	Bal.

**Table 3:** The mechanical properties—UTS (Ultimate tensile strength), YP (yield point) elongation, hardness and density of printed and AISI 316L stainless steel.

Material	UTS (MPa)	YP (MPa)	Elongation (%)	Hardness (HV)	Density (gr/cm^3^)
Printed 316L	552 ± 11	364 ± 17	42 ± 1	196 ± 5	7.6 ± 0.3
AISI 316L	752 ± 3	695 ± 3	37 ± 1	275 ± 9	7.8 ± 0.2

**Table 4:** Corrosion rate of tested alloys as obtained by Tafel extrapolation from potentiodynamic polarization curves.

Material	*E*_corr_ (v)	*I*_corr_ (μA/cm^2^)	Corrosion Rate (mmpy)	*E*_break_ (v)
Printed 316L	−0.25 ± 0.02	0.48 ± 0.12	0.005 ± 0.001	0.47 ± 0.03
AISI 316L	−0.21 ± 0.02	0.09 ± 0.004	0.001 ± 0.0003	0.18 ± 0.004

**Table 5:** Corresponding fitting parameters for the EIS analysis shown in Figure 9.

Material	R1 (Ohm)	Q1 (F·s^a−1^)	a	R2 (Ohm)
Printed 316L	15.6	7.31 × 10^−5^	0.705	92,178
AISI 316L	16.98	6.07 × 10^−5^	0.825	83,863

## Data Availability

Data sharing is not applicable to this article.
